# Genomic prediction for the Germplasm Enhancement of Maize project

**DOI:** 10.1002/tpg2.20267

**Published:** 2022-10-24

**Authors:** Anna R. Rogers, Yang Bian, Matthew Krakowsky, David Peters, Clint Turnbull, Paul Nelson, James B. Holland

**Affiliations:** ^1^ Program in Genetics North Carolina State Univ. Raleigh NC 27695 USA; ^2^ Bayer Crop Science 700 Chesterfield Pkwy W Chesterfield MO 63017 USA; ^3^ USDA–ARS Plant Science Research Unit and Dep. of Crop and Soil Sciences, North Carolina State Univ. Raleigh NC 27695 USA; ^4^ USDA–ARS Plant Introduction Research Unit, Iowa State Univ. Ames IA 50011 USA; ^5^ USDA–ARS Plant Science Research Unit and Dep. of Crop and Soil Sciences and NC Plant Sciences Initiative, North Carolina State Univ. Raleigh NC 27695 USA

## Abstract

The Germplasm Enhancement of Maize (GEM) project was initiated in 1993 as a cooperative effort of public‐ and private‐sector maize (*Zea mays* L.) breeders to enhance the genetic diversity of the U.S. maize crop. The GEM project selects progeny lines with high topcross yield potential from crosses between elite temperate lines and exotic parents. The GEM project has released hundreds of useful breeding lines based on phenotypic selection within selfing generations and multienvironment yield evaluations of GEM line topcrosses to elite adapted testers. Developing genomic selection (GS) models for the GEM project may contribute to increases in the rate of genetic gain. Here we evaluated the prediction ability of GS models trained on 6 yr of topcross evaluations from the two GEM programs in Raleigh, NC, and Ames, IA, documenting prediction abilities ranging from 0.36 to 0.75 for grain yield and from 0.78 to 0.96 for grain moisture when models were cross‐validated within program and heterotic group. Predicted genetic gain from GS ranged from 0.95 to 2.58 times the gain from phenotypic selection. Prediction ability across program and heterotic group was generally poorer than within groups. Based on observed genomic relationships between GEM breeding lines and their tropical ancestors, GS for either yield or moisture would reduce recovery of exotic germplasm only slightly. Using GS models trained within program, the GEM programs should be able to more effectively deliver on its mission to broaden the genetic base of U.S. germplasm.

## Core Ideas

- The Germplasm Enhancement of Maize (project is a public–private cooperative breeding project.
- The GEM project aims to enhance the genetic diversity of temperate maize.
- Until now, the GEM program has relied on phenotypic evaluations for selection decisions.
- We established a genomic prediction model using historical evaluation data from the GEM project.
- Results suggest that genomic prediction could increase genetic gain in the GEM project.

**Abbreviations**
BLUE: best linear unbiased estimate
GBLUP: genomic best linear unbiased predictor
GEM: Germplasm Enhancement of Maize
GS: genomic selection
IDV: identity variance structure
LAMP: Latin American Maize Project
NSS: nonstiff stalk
PVP: plant variety protection
SNP: single‐nucleotide polymorphism
SS: stiff stalk

## 1 INTRODUCTION

In the United States, maize (*Zea mays* L.) is the most important feed grain crop, with commercial hybrid cultivars produced by private‐sector companies. Commercial breeding programs are mostly closed genetic systems with extensive recycling of high‐performing lines (Mikel & Dudley, 2006) resulting in limited introgression or incorporation from outside breeding pools. This leads to concerns about genetic vulnerability to environmental changes, abiotic stresses, emergence of new pathogens and invasive insect species (Goodman et al., 2015; Pollak, 2003). Private‐sector germplasm in the United States represents reduced genetic variation relative to the global diversity of maize (Feng et al., 2006; Nelson & Goodman, 2008; Nelson et al., 2016; Van Heerwaarden et al., 2012).

At the metapopulation level, maize diversity is partitioned into groups corresponding to geographic origin, breeding history, and end use. Publicly available inbreds used in the United States group into at least five major genetic subpopulations including stiff stalk (SS), nonstiff stalk (NSS), tropical and subtropical, popcorn, and sweet corn (Liu et al., 2003). Breeding between these primary groups results in an additional admixed group of individuals that do not fit neatly into one of the primary groups (Liu et al., 2003). In addition, European Flints, widely used in Europe, have diverged substantially from their American progenitors (Rebourg et al., 2003). Genetic diversity can be enhanced by breeding between these primary groups, but care must be taken to not disrupt heterotic patterns that permit efficient testing of hybrid combinations. Most commercial inbred lines developed in the United States are derived from crosses within heterotic groups, often recycling important parental lines (Mikel & Dudley, 2006).

Genetic studies of elite germplasm have demonstrated that subpopulations in localized breeding programs have less diversity and higher linkage disequilibrium than the global maize metapopulation (Ching et al., 2002; Duvick et al., 2004; Yan et al., 2009). Heterotic groups have become increasingly separated over time, with some reduction in genetic diversity among elite inbreds with high genetic contributions to current breeding populations (Ching et al., 2002; Hufford et al., 2012; Romay et al., 2013). Concerns regarding decreases in genetic variability in these closed breeding pools prompted initiation of the Latin American Maize Project (LAMP), which evaluated 12,000 accessions from its 12 participating countries and was the first international project to systematically evaluate the global resources of a major crop (Pollak, 2003; Salhuana et al., 1998). The LAMP and related studies identified populations from Latin America that might be useful for both increasing the genetic variation and the yield potential of U.S. maize (Holland & Goodman, 1995; Holland et al., 1996; Holley & Goodman, 1988; Salhuana et al., 1998). Tropical maize harbors greater genetic diversity than temperate maize (Liu et al., 2003; Pollak, 2003; Tallury & Goodman, 2001) and is a source of alleles for improving yield and resistance to diseases that are absent in the elite temperate maize breeding pool (Goodman & Brown, 1988; Goodman et al., 2015; Nelson et al., 2006; Pollak, 2003; Tallury & Goodman, 2001). However, extracting useful alleles from exotic and unadapted sources through germplasm introgression breeding efforts is often a hindrance to short‐term genetic gain.

Building on the success of the LAMP Project, the Germplasm Enhancement of Maize (GEM) program was created to organize efforts of increasing diversity of U.S. maize germplasm in a public–private cooperative between the USDA's Agricultural Research Service (USDA–ARS), universities, and industry. The GEM program seeks to broaden the genetic base of U.S. germplasm by introgressing exotic accessions into elite proprietary germplasm provided by the private‐sector GEM cooperators and to release lines that can be readily integrated into elite temperate breeding programs. This ‘prebreeding’ step is intended to overcome the major adaptation barriers of the exotic germplasm. The program is spearheaded by two USDA projects located in Ames, IA (which mostly develops 25% exotic inbreds) and in Raleigh, NC (which mostly develops 50% exotic inbreds). Since the program's inception in 1993, 334 GEM lines have been released: 191 from Iowa, 124 from North Carolina, and 19 from other public entities (Supplemental Table S1).

**Core Ideas**

- The Germplasm Enhancement of Maize (GEM) project is a public–private cooperative breeding project.
- The GEM project aims to enhance the genetic diversity of temperate maize.
- Until now, the GEM program has relied on phenotypic evaluations for selection decisions.
- We established a genomic prediction model using historical evaluation data from the GEM project.
- Results suggest that genomic prediction could increase genetic gain in the GEM project.

**FIGURE 1:**
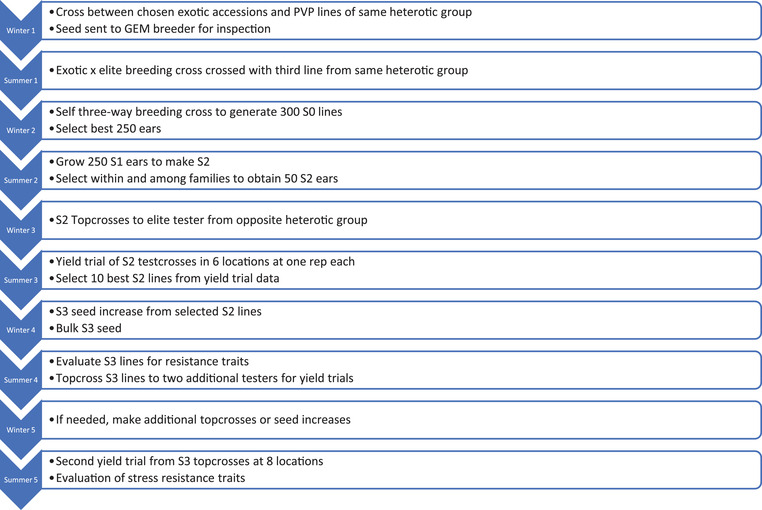
Schematic of current Iowa Germplasm Enhancement of Maize (GEM) program breeding practice following modified‐pedigree method. The North Carolina program follows a similar protocol but more often uses biparental crosses rather than three‐way crosses to initiate breeding populations

The GEM program breeding efforts have followed a modified pedigree method to develop lines from crosses between elite material made available by private cooperators and exotic germplasm accessions identified through public programs (Jumbo et al., 2011). The GEM program uses phenotypic selection, both within and among early inbreeding generation lines per se in summer nurseries and among topcross hybrids, created by crossing GEM line selection candidates to elite testers in multiple‐environment yield trials. Some of the nursery work and yield trial testing is provided to GEM in the form of in‐kind donations from private‐sector cooperators. A typical GEM breeding workflow results in the public release of S_2_– and S_3_–derived lines of 25–50% exotic derivation (Figure 1). The original protocol was to develop breeding lines from crosses between exotic accessions chosen by the GEM coordinators to elite adapted proprietary inbreds chosen by private industry partners. More recently, lines with expired plant variety protection (PVP) certificates or previously released GEM lines have also been used as parents of breeding crosses. In the Ames, IA, program, the F_1_ breeding cross is then crossed to a third proprietary line within the same heterotic group to create a three‐way cross of 25% exotic parentage. The North Carolina GEM program typically initiates selection within segregating populations with 50% exotic parentage. In the third season, an S_0_ bulk is grown in a temperate nursery and plants with acceptable adaptation are selfed to generate ∼300 ears per population. The selected S_1_ ears are then planted ear‐to‐row, selfed, and subjected to per se selection for acceptable flowering, ear height, lodging, disease resistance, and ear production. Per se selection is done within and among S_1_ families to select 50–100 desirable S_2_ ears per family to topcross with an elite tester from the complementary heterotic group. First stage topcross yield trials are conducted at approximately six locations with one replication per location. Selection among topcross hybrids is for higher grain yield and lower grain moisture and lodging. The best 15–20% of S_2_ lines are then selected and increased in bulk to form S_2:3_ lines. These lines are also evaluated for agronomic quality and disease and pest resistance as lines per se and topcrossed to an additional tester from the complementary heterotic group. The topcrosses are evaluated at 10–18 locations, and the best lines are coded and released as S_2:4_ lines for public distribution via the North Central Regional Plant Introduction Station.

During the initial years of GEM, all breeding crosses within GEM were derived from crosses between exotic accessions and one or two elite proprietary inbreds, where the elite‐adapted parents were provided by one of several private‐sector GEM cooperators. To maintain intellectual property, information about the commercial lines used as parents of GEM lines was never revealed beyond their heterotic group origin (SS or NSS, groups). The inability to directly genotype commercial parents on one side of the pedigree and the use of genetically heterogeneous exotic populations on the other side of the pedigree has limited the scope of genetic analysis that can be conducted with GEM lines. In recent years, however, the GEM project has shifted toward recycling GEM lines and using expired PVP lines (Mikel & Dudley, 2006; Nelson et al., 2008; White et al., 2020) as parents of breeding populations. This shift has expanded the opportunities for genetic study with GEM breeding lines and populations including the implementation of genomic selection (GS) where parental data is important to facilitate low‐cost genotyping with imputation. Integrating GS into the GEM breeding protocol may improve genetic gain by shortening cycle time, increasing selection intensity by expanding the number of selection candidates beyond the limit imposed by phenotyping resources, and allowing for optimization of crossing within a population (Guo et al., 2019; Lorenz et al., 2011; Sorrells, 2015).

This study is the first to examine a GEM project breeding population using genetic data with the objectives of (a) evaluating prediction ability of GS models for grain yield and grain moisture topcross combining ability trained and evaluated within heterotic groups of the two GEM programs (Iowa and North Carolina), (b) evaluating prediction ability of models trained in one combination of program and heterotic group and tested across programs and groups, and (c) predicting the impact of GS on the proportion of exotic germplasm maintained in selected lines.

## 2 MATERIALS AND METHODS

### 2.1 Phenotypic data

Germplasm Enhancement of Maize program breeders supplied the yield trial data that were used in model training. Some of the data were generated and donated to GEM by cooperator companies as part of their generous in‐kind support for the GEM project. Some of the data were generated within the Iowa and North Carolina GEM programs. Yield trial locations were coded uniquely within each year in the dataset to respect GEM cooperator confidentiality; location–year combinations were fit as environments in analysis models. The Iowa and North Carolina programs both used S_2_ GEM breeding line topcross testing schemes, but the two programs operate independently in terms of germplasm, testers, and evaluation environments. For this reason, data sets for the two programs were treated separately throughout analysis.

For the Iowa program, yield trials comprised data from 2014 through 2019 from locations throughout the U.S. Midwest, comprising 12,373 observations across 45 environments (Supplemental File S1). Across all years, a total of 13 testers was used, which mostly aligned with the complementary heterotic group in the breeding population, although sometimes, lines were crossed to both SS and NSS testers. Data were from single‐replication per location trials with checks replicated across experiments, locations, and years. After removing missing data, 11,717 records for grain yield and moisture remained in the Iowa dataset. Of these, 898 were records of checks, with the remaining 10,819 observations on 1,762 unique experimental GEM lines, some of which were crossed to multiple testers. These lines represented 27 full‐sib families from two‐ and three‐way crosses with some lines being used as parents in multiple populations. Later comparison of genomic relationships between GEM progeny lines and their ancestors revealed discrepancies between two breeding families and their putative parents; these two families were dropped from further analyses, resulting in 25 breeding families in the Iowa program data set (Table 1).

**TABLE 1:** Families composing the Ames, IA, Germplasm Enhancement of Maize (GEM) breeding program yield trial data set used for genomic model calibration and testing

Cross code	Family	No. hybrids	No. lines	Mean grain yield	Mean grain moisture
				Mg ha^−1^	%
1	3IIH6 //GEMN‐0173/KO679Y	67	67	10.9	19.3
2	CML341/DJ7//PHJ40	66	57	9.9	17.7
3	GEMN‐0140/GEMN‐0097//GEMN‐0205	177	177	11.1	19.3
4	GEMN‐0179/GEMN‐0205	45	45	11.5	19.8
5	GEMN‐0192/GEMN‐0152	65	65	8.8	19.8
6	GEMN‐0193/GEMN‐0179	70	70	9.7	22.6
7	GEMN‐0239//GEMN‐0097/Ki14	54	54	10.9	20.0
8	GEMS‐0113/GEMS‐0162	31	31	9.9	20.1
9	GEMS‐0115/GEMS‐0222	56	55	11.0	16.9
10	GEMS‐0147/GEMS‐0227//GEMS‐0219	39	39	11.4	16.1
11	GEMS‐0162/PHP38//GEMS‐0227	141	141	11.7	19.8
12	GEMS‐0219//KO679Y/GEMS‐0030	90	90	11.1	16.4
13	GEMS‐0219/GEMS‐0227	57	53	11.4	16.8
14	GEMS‐0226//GEMS‐0182/GEMS‐0227	31	31	12.1	22.2
15	GEMS‐0227//LH195/KO679Y	91	91	9.9	20.1
16	GEMS‐0227/GEMS‐0189	106	106	10.8	17.5
17	GEMS‐0227/PHP38	67	67	12.6	20.2
18	Ki21/DJ7//GEMS‐0115	49	49	10.9	18.5
19	KO679Y/GEMS‐0115//GEMS‐0162	43	43	10.9	20.9
20	LH57/CML373//GEMN‐0155	66	36	10.1	20.4
21	LH82/KO679Y	81	81	10.2	19.2
22	LH82/KO679Y//GEMN‐0205	67	67	10.5	22.0
23	NS1/GEMS‐0149//PHJ40	60	49	9.5	18.4
24	PHB47/CML373//GEMS‐0162	88	88	11.2	21.9
25	PHP38//GEMS‐0227/Ki14	67	67	11.7	22.1
CHK	Check	20	20	12.3	19.6
GEM	GEMCheck	8	4	12.1	19.8

*Note*. Number of lines refers to the number of unique progeny lines from the cross evaluated for yield. Number of hybrids is the number of unique progeny topcrosses (progeny line crossed to an individual tester) evaluated. Mean grain yields for each family are best linear unbiased estimates adjusted for differences in environment effects. Check group includes commercial hybrids and crosses among private sector lines. The GEM check group includes topcrosses among released GEM lines and between released GEM lines and private sector lines. Cross codes correspond to codes displayed in Figure 2a.

For the North Carolina program, yield trials comprised data from 2017 through 2019 from locations throughout the southern United States. The dataset contained 6,021 observations for yield (Supplemental File S2), representing 118 combinations of 18 experiments and 31 coded environments. For North Carolina, a single tester was used for each heterotic group such that all SS lines were crossed to a single NSS tester, LH283 × LH287, and all NSS lines were crossed to a single SS tester, LH132 × PHG39. Data were from single replication per location with checks replicated across years and experiments. After removing records missing trait data, there were 5,670 observations for yield and 5,578 for moisture in the North Carolina dataset. In this dataset, 589 distinct GEM lines representing 19 breeding families were present (Table 2).

**TABLE 2:** Families composing the Raleigh, NC, Germplasm Enhancement of Maize (GEM) breeding program yield trial data set used for genomic model calibration and testing

Cross code	Family	No. lines	Mean grain yield	Mean grain moisture
			Mg ha^−1^	%
1	89291:S(LH195)	34	8.1	16.2
2	CA00370/GEMN‐0048	19	8.0	18.3
3	CML422:N(DK_HBA1)	42	7.7	18.3
4	CML449:N(PHG47)	59	8.3	16.2
5	CML494:S(PHP38)	18	8.4	18.2
6	GEMN‐0097/GEM‐0048	64	8.6	15.4
7	GEMN‐0104/GEMN‐0048	25	8.0	18.8
8	GEMN‐0104/GEMN‐0097	71	8.3	18.9
9	GEMN‐0124/GEMN‐0048	22	8.6	17.4
10	GEMN‐0124/GEMN‐0095	26	7.9	18.0
11	GEMN‐0124/GEMN‐0135	57	8.9	19.1
12	GEMN‐0135/GEMN‐0104	27	7.6	18.0
13	GEMS‐0027/GEMS‐0113	38	8.3	16.9
14	GEMS‐0027/GEMS‐0200	38	8.6	16.7
15	GEMS‐0220/GEMS‐0032	33	8.4	16.9
16	KO679Y:S(LH195)	21	7.9	16.8
17	La Posta Seq C7 F71‐1‐1‐1‐2‐B*3/LH193	58	8.9	18.8
18	La_Posta_Seq_C7_F64‐1‐1‐1‐1‐B‐B‐B/LH193	47	9.0	16.9
19	La_Posta_Seq_C7_F64‐1‐1‐1‐1‐B‐B‐B/PHP38	24	9.0	17.7
CHK	Check	16	10.3	17.5

*Note*. Number of lines refers to the number of unique progeny lines from the cross evaluated for yield. In the North Carolina GEM program data set, each experimental GEM line was crossed to a single tester, so the number of lines tested per family is equal to the number of hybrids tested. Mean grain yields for each family are best linear unbiased estimates adjusted for differences in environment effects. Check group includes commercial hybrids and crosses among private sector lines. Cross codes correspond to codes displayed in Figure 2b.

Yield trial experiments (sets) refer to a group of GEM line topcrosses within a given year replicated across multiple locations but never replicated across years. For GS model training, we combined data across experiments and years, introducing significant imbalance in the final dataset. Check hybrids were replicated across environments, permitting combined analysis across all environments within each program.

### 2.2 Genomic data

High‐density single‐nucleotide polymorphism (SNP) data for 100 parental lines of GEM progenies were generated by Bayer Crop Science using a proprietary genotyping array. The SNPs for parental samples went through a quality control process to ensure marker quality, abundance, and coverage in the genome. A subset of over 40,000 markers was then selected and imputed using a proprietary reference panel and imputation algorithm. The progeny lines were genotyped using a low‐density sequencing technology. Imputation was then performed to bring low‐density progeny samples to SNP density equal to the parental lines using a proprietary Bayer reference panel and genetic map and BEAGLE 5.0 with default parameter settings (Browning & Browning, 2007; Browning et al., 2018). After imputation, no missing data were present. Data from imputed progeny and parental lines were then used to create an additive genomic relationship matrix (**A**) for use in GS modeling with the R package AGHmatrix (Amadeu et al., 2016) using the VanRaden parameterization based on observed allele frequencies (VanRaden, 2008). The genomic relationship matrix is in Supplemental File S3; R codes to display the matrix as a heatmap and perform principal components analysis of the relationship matrix are in Supplemental Files S4 and S5.

### 2.3 Statistical analyses

After removing lines lacking genotype data or that were filtered because of discrepancies between their genomic and pedigree relationships to parents, we had both genotype and phenotype data on 1,491 lines from the Iowa program and 589 lines from the North Carolina program. Because of differences between the Ames, IA, and Raleigh, NC, GEM programs, the two sets of trials were treated as separate datasets and analyzed with different models. Analysis of data from each program followed a two‐stage approach outlined in general here and then in detail for each program in subsequent sections. First, best linear unbiased estimators (BLUEs) for the mean topcross breeding values of each GEM line were estimated as marginal means across environments adjusting for the effects of testers (for the Iowa program). Then, genomic best linear unbiased predictors (GBLUPs) for each GEM line were computed using the genomic relationships from the additive genomic relationship matrix (**A**) in a general combining ability model. Although it is possible to incorporate specific combining ability effects into genomic prediction models (Kadam et al., 2016), doing so requires genomic information on the testers, which were not available in this study because some testers were commercial lines not available for genetic study.

#### 2.3.1 Stage 1: Estimated breeding values

In the first step, BLUEs for each GEM line were estimated using ASReml‐R 4.0 (Butler et al., 2017) using the following model fit to the Iowa data:

$$y_{ijk}=\mu +E_{i}+T_{j}+G_{k}+\varepsilon_{ijk}$$

where *y_ijk_* is the grain yield or moisture value of line *k* crossed to tester *j* in environment *i*, μ is the overall mean yield, *E_i_* is the random effect of environment *i*, *T_j_* is the random effect of tester *j* (with check hybrids given a common level for tester), *G_k_* is the fixed effect associated with GEM line or check hybrid *k*, and ε*_ijk_* is the residual effect, which includes confounded effects of experimental error and line × environment interaction because of the lack of replication within environments.

A different model was fit to the North Carolina program data because of the different treatment and experimental design structures. In this program, each line was crossed to one tester with different testers used for the two different heterotic groups. Therefore, we cannot separately estimate the confounded GEM line and tester effects. For North Carolina, BLUEs for each line were generated using the following model:

$$y_{ijk}=\mu +E_{i}+S_{j}+ES_{ij}+G_{k}+\varepsilon_{ijk}$$

where *y_ijk_* is the grain yield or moisture value of line *k* in environment *i*, μ is the overall mean yield, *E_i_* is the random effect of environment *i*, *S_j_* is the random effect of experiment *j*, ES*_ij_* is the random effect of the interaction of environment *i* and experiment *j*, *G_k_* is the fixed effect associated with GEM line or check hybrid *k*, and ε*_ijk_* is the residual effect. Environments are coded and their relationships across years are unknown. Supplemental Files S6, S7, S8, and S9 contain R code for this analysis.

The BLUEs for each GEM line were obtained from these models as follows:

$${\hat{Y}}_{k..}=\mu +{\hat{G}}_{k}$$

These BLUEs for grain yield and grain moisture were used as input phenotypes for genomic prediction models in training sets and as ‘observed breeding values’ in test sets for measuring genomic prediction ability.

#### 2.3.2 Stage 2: Genomic estimated breeding values

Check hybrids were removed from the stage one BLUEs data sets, then the following model was fit to obtain GBLUPs of GEM line breeding values using the additive genomic relationship matrix (**A**):

$${\hat{Y}}_{k..}=\mu +{\hat{G}}_{k}+\varepsilon_{k}$$

where the ‘observed’ values ${\hat{Y}}_{k..}$are the estimated breeding values for either grain yield or grain moisture obtained as BLUEs from the Stage 1 analyses, μ is the grand mean, ${\hat{G}}_{k}$ is the random effect of GEM line *k* modeled using the additive genomic relationship matrix (**A**) such that $G_{i}∼N\left(0,A\sigma_{G}^{2}\right)$, and ε*_k_* is the residual effect of the estimated breeding value. The GBLUPs from this model are predictions of GEM line breeding values. The Stage 2 analyses were performed with ASReml‐R 4.0 (Butler et al., 2017).

### 2.4 Measurement of prediction ability

#### 2.4.1 Prediction across groups

We evaluated prediction ability using six distinct training sets:

1. Iowa SS GEM lines (N = 913 lines)
2. Iowa NSS GEM lines (N = 578 lines)
3. All Iowa lines (both SS and NSS, N = 1,491 lines)
4. North Carolina SS GEM lines (N = 182 lines)
5. North Carolina NSS GEM lines (N = 407 lines)
6. All North Carolina lines (both SS and NSS, N = 589 lines)

For each training set 1, 2, 4, and 5, the lines in the other five groups represented test sets, and we predicted line values in all test sets. For training sets 3 and 6, the lines in the two groups from the other program constituted the test set. We evaluated prediction ability within program and heterotic group, which is most relevant to making breeding decisions (i.e., the best lines within groups will be selected and intermated within groups to create new breeding populations). Prediction ability was measured as the correlation between the GBLUPs and BLUEs within a program and heterotic group.

#### 2.4.2 Prediction within groups

We also performed tenfold cross‐validation of prediction ability within program and heterotic group by splitting each of the six complete training sets into 10 disjoint folds of approximately equal size (maximum sample size difference of no more than one). The prediction model was fit to the data from nine of the folds, with data from the 10th fold held out to serve as a test set. Prediction ability was measured as the correlation between GBLUPs and BLUEs within heterotic group of the test set. For example, when the training set included both SS and NSS lines from the Iowa program, we evaluated prediction ability separately within the 10% of Iowa SS test set lines and the 10% of Iowa NSS test set lines. This process was repeated, holding out a different fold as test set each time, and the correlation between BLUEs and GBLUPs within the test set was averaged over the 10 folds. Supplemental Files S10, S11, S12, and S13 contain R code to perform these analyses.

#### 2.4.3 Prediction across years

We investigated the possibility of genomic prediction using training data from multiple years to predict breeding values in a different year. We used only the Iowa data set for this analysis, as it had data from 6 yr. We created six pairs of training and test data sets by separating the data from a single year as a test set, with data from all other years as its paired training set. There was a maximum of three lines in common between a pair of training and test sets. We repeated the process of Stage 1 analysis to obtain line BLUEs within each training and test set separately, fit the GBLUP model to the training set, and then measured the correlation between the predicted and observed values of the test set lines for each test set (Supplemental Files S14 and S15).

### 2.5 Heritability and genetic gain

Genomic heritability of GEM line breeding values after adjusting for tester effects was computed from the variance components estimates of the Stage 2 GBLUP models as follows:

$${\hat{h}}^{2}=\frac{{\hat{\sigma }}_{A}^{2}}{{\hat{\sigma }}_{A}^{2}+{\hat{\sigma }}_{e}^{2}}$$

where ${\hat{\sigma }}_{A}^{2}$ is the variance component from the random effects of the GEM lines fit using the **A** matrix, and ${\hat{\sigma }}_{e}^{2}$ is the residual variance.

Similarly, heritability corresponding to phenotypic selection among line topcross values without the use of genomic information was computed using the following:

$${\hat{h}}^{2}=1-\left(\frac{\bar{\mathrm{SE}}}{2{\hat{\sigma }}_{G_{\mathrm{IDV}}}^{2}}\right)$$

where $\bar{\mathrm{SE}}$ is the average standard error of pairwise differences between topcross or general combining ability BLUPs from a model fitting the random genetic component using an identity variance structure (IDV), and ${\hat{\sigma }}_{G_{\mathrm{IDV}}}^{2}$ is the genetic variance component from that model (Cullis et al., 2006). These estimates were obtained from an analysis of the data sets excluding check cultivars. We estimated genomic and phenotypic heritability across heterotic groups within programs and also within each heterotic group and program combination (Supplemental Files S4, S5, S6, and S7).

Predicted genetic gain based on phenotypes only is formulated as a response to selection as follows:

$$R=ih\sigma_{A}$$

where *R* is the response to selection, *i* is the selection intensity, *h* is the square root of narrow‐sense heritability, and σ_A_ is the additive genetic variance (Falconer & Mackay, 1996). Response to GS can be formulated in terms of the accuracy of genomic predictions as follows:

$$R=ir_{A}\sigma_{A},$$

where *r*_A_ is the prediction accuracy (Lorenz, 2013).

Prediction ability (PA) is related to prediction accuracy as follows (Estaghvirou et al., 2013):

$$r_{A}=\mathrm{PA}/h$$

Assuming the selection intensity remains the same between phenotypic and GS, we can use these relationships to compute the predicted gain from GS relative to gain from phenotypic selection as follows:

$$\frac{R_{\mathrm{GS}}}{R_{\mathrm{phenotypic}}}=\frac{ir_{A}\sigma_{A}}{ih\sigma_{A}}=\frac{r_{A}}{h}=\frac{\mathrm{PA}/h}{h}=\frac{\mathrm{PA}}{{\hat{h}}^{2}}$$

where PA is the genomic prediction ability and ${\hat{h}}^{2}$ is heritability estimated using the IDV model based on phenotypes only. This equation involves only the mean prediction ability correlation estimate in the numerator and the phenotypic heritability estimate in the denominator. Errors in estimation of these two parameters contribute to errors in our estimates of relative genetic gain. This calculation was made for selection within each combination of program and group using the corresponding within‐group prediction ability and heritability estimates.

### 2.6 Parentage analysis

Parent and grandparental lines were grouped into three categories: expired PVP temperate lines, previously released GEM lines, and tropical lines. For the 19 families (1,076 lines) derived from crosses involving more than one group, we estimated the mean genomic relationship coefficient between each progeny line and its ancestor lines within each parental group. For each family we separately computed expected genomic relationships between each progeny and its parental groups as: **G**_sub_ **w**, where **G**_sub_ is a submatrix of the overall genomic relationship matrix including only the rows and columns corresponding to the ancestors (parents or grandparents), and **w** is a vector of expected pedigree weights (0.25 for grandparents and 0.5 for parents). This method accounts for the inbreeding of the parents and relationships among the parents. We computed the deviation between the observed and expected parental group relationship coefficients separately for each family then averaged over families (Supplemental Files S4 and S5).

We estimated the correlation between trait BLUE values and parental group relationships within each family. We also estimated the correlation between trait GBLUP values and parental group relationships within each family. Finally, within each program and group, we estimated the effect of selection on progeny genomic relationships with the parental germplasm groups. This was done by estimating the difference in mean genomic relationship coefficient to each of the parental germplasm groups for the 10% of lines with best GBLUP values for each trait (higher grain yield or lower grain moisture) compared with all lines within each program and heterotic group (Supplemental Files S4 and S5).

## 3 RESULTS AND DISCUSSION

### 3.1 Trait distributions

**FIGURE 2:**
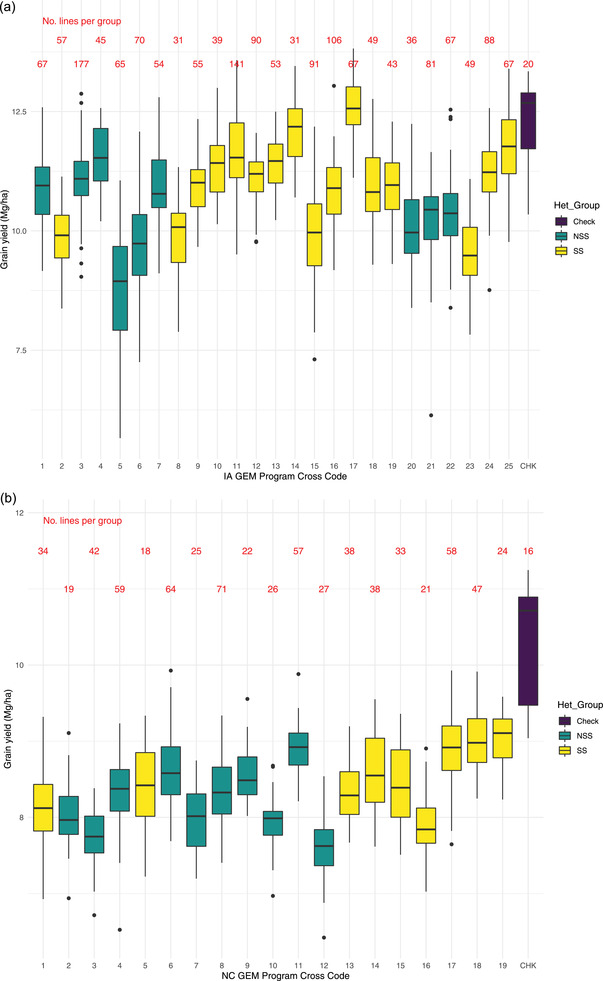
Box plots representing the distribution of topcross yield means of (a) Iowa Germplasm Enhancement of Maize (GEM) and (b) North Carolina GEM program breeding families and check hybrids (CHK). The number of lines tested per family is displayed in red font above boxes. Family codes on horizontal axis in (a) correspond to codes in Table 1; codes in (b) correspond to codes in Table 2. SS, stiff stalk; NSS, nonstiff stalk

Mean topcross hybrid grain yield varied widely among GEM lines in both programs. Family mean yields ranged from 8.8 to 12.6 Mg ha^−1^ in the Iowa program, and an even greater range of yield performance was observed among individual GEM lines (Figure 2a, Table 1; Supplemental Table S2). The highest yielding GEM family in the Iowa program was GEMS‐0227/PHP38 (Cross code 17 in Figure 2a); the mean grain yield across all lines within this family was 0.3 Mg ha^−1^ greater than the mean of the commercial checks (Table 1). Environment marginal mean predictions (BLUPs of the random environments adjusted for differences in the hybrids tested in different environments) ranged from 6.7 to 13.4 Mg ha^−1^ (Supplemental Figure S1), demonstrating a wide range of productivity among the testing environments.

The range of grain yield variation among families in the North Carolina program data set was smaller, with family means ranging from 7.6 to 9.0 Mg ha^−1^ (Figure 2b, Table 2; Supplemental Table S3). None of the North Carolina program family means were competitive with the commercial checks, which had a mean yield of 10.3 Mg ha^−1^. This likely is due to the higher proportion of exotic germplasm in these lines. The range of environment marginal mean yield predictions was much greater in the North Carolina data set, however, ranging from 4.0 to 12.1 Mg ha^−1^ (Supplemental Figure S2), reflecting the variability in growing conditions and potential for stress in southern maize production areas.

Grain moisture also varied considerably among families within both programs: from 161 to 226 g kg^−1^ in the Iowa program and from 154 to 191 g kg^−1^ in the North Carolina program (Tables 1 and 2; Supplemental Figures S3 and S4). Importantly, approximately half of GEM families in both programs had mean grain moisture lower than their respective check means (Tables 1 and 2; Supplemental Figures S3 and S4). This result indicates that hybrids containing substantial proportions of tropical germplasm can be developed with maturity and grain dry‐down characteristics, which is comparable with current commercial hybrids.

The phenotypic correlation between grain yield and grain moisture on an individual plot basis was *r* = 0.07 (*p* < .0001) in the Iowa data set and *r* = 0.28 (*p* < .0001) in the North Carolina data set. The correlations between line topcross breeding value BLUEs for the two traits were even smaller and not significantly different from zero in Iowa and *r* = 0.11 (*p* < .01) in North Carolina (Supplemental File S9). The correlations on the basis of adjusted line mean values are expected to be closer to the genetic correlations; therefore, we interpret these results as indicating low genetic correlations between grain yield and moisture in these data.

### 3.2 Heritability estimates

Phenotypic‐based estimates of heritabilities were generally high for the Iowa program, ranging from .59 to .65 for yield and .77 to .82 for moisture within and across groups of the Iowa program (Table 3). The phenotypic heritabilities of the North Carolina program were substantially lower (from .26 to .32 for yield and .48 to .61 for moisture). We believe that this partly is due to the structure of the North Carolina data set, in which check hybrids constitute 16% of observations and provide substantial connectivity across environments but are excluded from the heritability estimates of the GEM lines.

**TABLE 3:** Estimates of additive genetic variance (${\hat{\sigma }}_{A}^{2}$) and genomic heritability (${\hat{h}}_{g}^{2}$) from genomic best linear unbiased predictors model, genotypic variance (${\hat{\sigma }}_{g}^{2}$) and phenotypic heritability (${\hat{h}}_{p}^{2}$) assuming independent genetic effects, and relative efficiency of genomic vs. phenotypic selection ($\frac{R_{\mathrm{GS}}}{R_{\mathrm{phenotypic}}}$) for grain yield and grain moisture within heterotic groups and programs of the Germplasm Enhancement of Maize program

		Grain yield					Grain moisture				
Program	Group	${\hat{\sigma }}_{A}^{2}$	${\hat{h}}_{g}^{2}$	${\hat{\sigma }}_{g}^{2}$	${\hat{h}}_{p}^{2}$	$\frac{R_{\mathrm{GS}}}{R_{\mathrm{phenotypic}}}$	${\hat{\sigma }}_{A}^{2}$	${\hat{h}}_{g}^{2}$	${\hat{\sigma }}_{g}^{2}$	${\hat{h}}_{p}^{2}$	$\frac{R_{\mathrm{GS}}}{R_{\mathrm{phenotypic}}}$
Iowa	NSS	0.71	.65	0.52	.65	1.08	2.34	.88	1.33	.82	0.95
	SS	0.52	.65	0.40	.59	1.27	1.00	.96	0.52	.77	1.25
	SS + NSS	0.56	.63	0.44	.62	–	1.43	.88	0.83	.81	–
North Carolina	NSS	0.23	.68	0.05	.26	2.58	0.33	.72	0.16	.61	1.34
	SS	0.05	.18	0.08	.32	1.13	0.18	.59	0.18	.48	1.64
	SS + NSS	0.19	.57	0.06	.29	–	0.28	.69	1.04	.55	–

*Note*. NSS, nonstiff stalk; SS, stiff stalk.

Genomic heritability estimates were typically greater than their corresponding phenotypic heritability estimates across traits and groups (Table 3), in some cases by a substantial amount (>.40 greater for grain yield in North Carolina NSS group). Genomic heritabilities can provide connectivity among experiments and environments through genomic relationships even in the absence of common entries, which likely results in the greater estimates of genomic heritability in the North Carolina data set in particular. However, one group deviated from this trend: the genomic heritability of yield in the North Carolina SS group was only .18, lower than its corresponding phenotypic heritability estimate of .32 (Table 3). We do not have a good explanation for this anomaly; the genomic heritability of moisture in this group is greater than its corresponding phenotypic heritability estimate, ruling out large‐scale genotyping or sampling handling errors.

### 3.3 Genomic prediction ability and population structure

**FIGURE 3:**
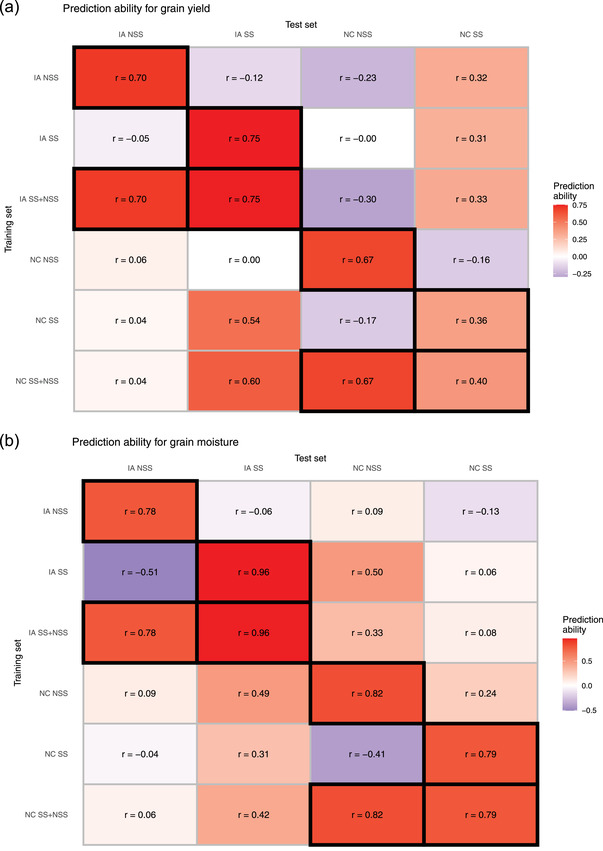
Heatmaps of prediction ability (correlations between observed line mean values and genomic prediction values) within and between programs and heterotic groups for (a) grain yield and (b) grain moisture. Prediction ability was measured as the mean correlation over 10 folds of cross‐validation within groups (displayed within dark borders) and as the single correlation value for prediction across groups

Models trained in one program and heterotic group and evaluated on independent samples of the same program and group had high prediction ability for yield in the Iowa program (*r* = 0.72–0.75) and the North Carolina NSS group (*r* = 0.67) but much lower in the North Carolina SS group (*r* = 0.37; Figure 3a; Supplemental Table S4). This latter group also had the lowest phenotypic and genomic heritability for yield (Table 3). Mean prediction ability within groups was better for grain moisture (ranging from 0.80 to 0.95; Figure 3b; Supplemental Table S5). Training on both heterotic groups within a program improved prediction ability only for yield within North Carolina SS group, illustrating the limited genetic connectivity between heterotic groups (Figure 3).

Training the genomic prediction model in one heterotic group to predict the other heterotic group within the same program resulted in negative prediction abilities for grain yield (ranging from −0.05 to −0.17; Figure 3a; Supplemental Table S4). Training on grain moisture in one heterotic group to predict the other heterotic group within the same program resulted in low to negative prediction abilities (*r* = −0.51 to 0.24; Figure 3b; Supplemental Table S5). These results confirm that heterotic group‐specific approaches should be favored in genomic prediction for yield in maize, congruent with the partitioning of distinct allele effects for grain yield across different heterotic groups in maize driven by both drift and selection for complementary alleles underlying heterosis (Giraud et al., 2017, 2014; Gerke et al., 2015).

We also evaluated prediction ability of models trained in one program and evaluated in the same heterotic group of the other program. These correlation values ranged from −0.23 to 0.54 for grain yield and from 0.09 to 0.31 for grain moisture. Surprisingly, for grain moisture, we found substantially higher prediction ability for models trained in North Carolina NSS and evaluated in Iowa SS or vice versa (*r* = 0.31–0.50; Figure 3) than when models were trained in the same heterotic group but evaluated across programs. This result may be due to chance associations between predicted and observed family means for moisture driven by the relatively small number of families in the North Carolina NSS group, as the within‐family prediction ability was significant (*p* < .05) in only one of the 25 test set families (Supplemental Files S16 and S17). Furthermore, we observed underdispersion of predicted values compared with observed values in these cases; with predicted values having 2% or lower variance relative to the observed values, indicating low reliability of these cross‐group and cross‐program predictions (Supplemental Table S5). Therefore, even though we identified some cases where prediction ability of genomic estimated breeding values held up across programs or heterotic groups, this result was not consistent, so we recommend developing and applying genomic prediction models within programs and groups.

We did not attempt to combine all data across both programs into a common training set because the Iowa and North Carolina data sets are completely unconnected in terms of environments and testers. Training models on BLUEs averaged over different sets of environments and testers would be expected to predict differences in the program means, but this difference may mostly be due to nongenetic effects; such predictions would not be expected to accurately rank breeding values within programs.

**FIGURE 4:**
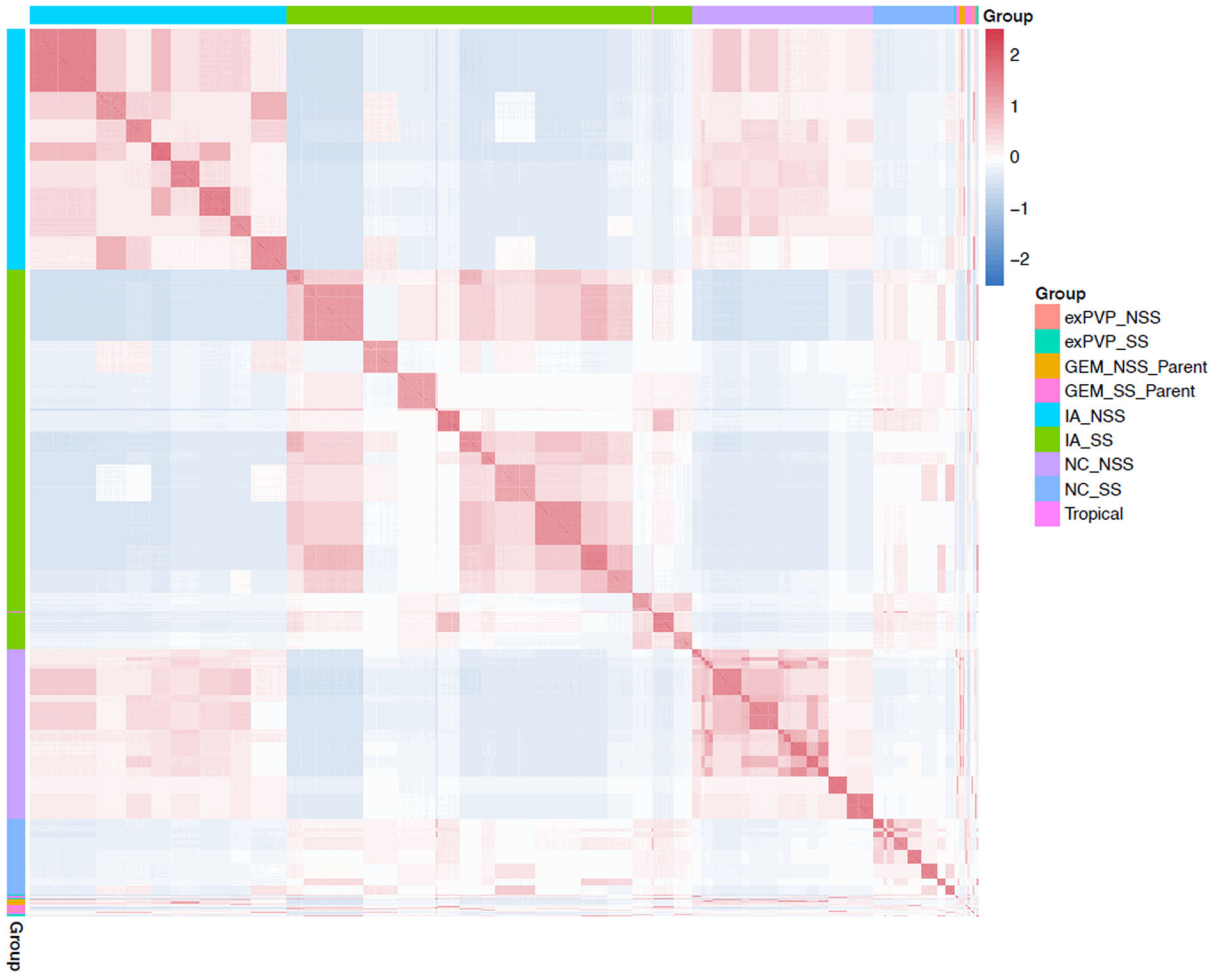
Heatmap of additive realized genomic relationship matrix of Germplasm Enhancement of Maize (GEM) lines. Program and heterotic group of each line is denoted by colored bars on top and left sides. SS, stiff stalk; NSS, nonstiff stalk; PVP, plant variety protection

**FIGURE 5:**
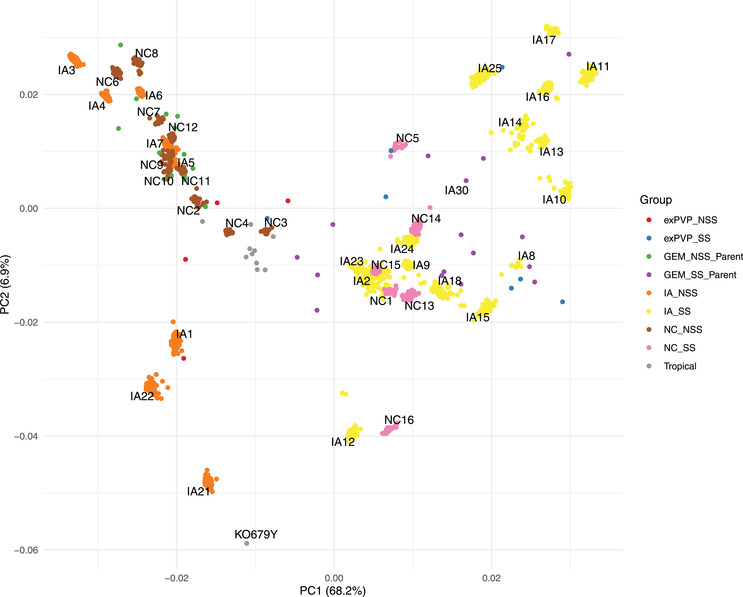
Plot of Germplasm Enhancement of Maize (GEM) lines colored according to their program and heterotic group on the first two principal components of the genomic relationship matrix. The GEM breeding families are labelled using the cross codes from Tables 1 and 2. Tropical founder line KO679Y is also labeled. SS, stiff stalk; NSS, nonstiff stalk; PVP, plant variety protection

The genetic differentiation between the Iowa and North Carolina programs is observable in the heat map representation of the combined genomic relationship matrix (Figure 4) and the plot of GEM lines on the first two principal components of the genomic relationship matrix (Figure 5). Blocks of full‐ and half‐sib families can be observed within programs, but low and even negative genomic relationship coefficients are common between the two programs (Figure 4).

Despite differences in the parental lines used by the two programs, we observed some genetic similarity between the Iowa and North Carolina NSS groups, perhaps driven by limited genetic variation of the temperate NSS parents used by the two programs. This is most clearly observed in the principal components plot (Figure 5), where it is apparent that differentiation is greater between heterotic groups than between programs and that there is substantial overlap between programs within heterotic groups. The first principal component is associated with 68% of variation in the relationship matrix and completely separates SS from NSS lines. The tropical parental lines group near zero on this axis, reflecting their limited representation in the genetic sample. The Iowa SS group has the widest spread on this axis and the North Carolina SS group clusters more tightly. The second principal component is associated with 7% of the variation and is driven in part by one unique tropical founder line, KO679Y, which is the only African parental line used in this study. The GEM breeding families derived from crosses with KO679Y all fall in the most negative end of the second principal component, clearly separated from the other breeding families. Although the tropical parents are mostly clustered in the center of the graph, the overall set of parental lines, including the expired PVP temperate parents, is spread across most of the range of these first two principal components (Figure 5). Finally, we note that the GEM family with highest mean yields (GEMS‐0227/PHP38) appears at the extreme upper right corner of the principal component plot, suggesting that it is more closely related to temperate SS germplasm than other families.

To quantify the among‐group differentiation, we estimated the mean genomic relationship coefficients within and between groups, finding that mean within‐group relationships were highest in the two NSS groups (0.50–0.52) and lower within the SS groups (0.27 for Iowa and 0.40 for North Carolina; Table 4). The Iowa and North Carolina NSS groups were also moderately related to each other, with mean between‐group relationship coefficient of 0.25. All mean relationships between heterotic groups were negative, demonstrating their strong differentiation (Table 4).

**TABLE 4:** Mean genomic relationship coefficients within and between programs and heterotic groups

		Iowa		North Carolina	
Program	Group	NSS	SS	NSS	SS
Iowa	NSS	0.52	−0.32	0.25	−0.18
	SS	–	0.27	−0.27	0.07
North Carolina	NSS	–	–	0.50	−0.13
	SS	–	–	–	0.40

*Note*. NSS, nonstiff stalk; SS, stiff stalk.

### 3.4 Prediction across years

One possible use of GS is to use historical data collected in previous years and potentially on different breeding families to predict the value of new families in future years. To model this application of GS, we evaluated prediction ability when all data form one year were held out as a test set, the GS model was trained on the other years combined, and the test set was predicted. There was almost no overlap of lines between years, so, in general, this was a test of prediction across distinct germplasm and environment sets. As expected, genomic prediction ability decreased substantially when an entire year was held out as the test set (Supplemental Table S6). Prediction ability for yield was not significant in two of the six cases and was negative (*r* = −0.30) when year 2015 was the test set. Prediction ability for yield ranged from 0.35 to 0.49 among the other 3 yr where it was significant. The same pattern held for grain moisture, with *r* = −0.64 for year 2015 test set and ranging from 0.31 to 0.96 among the 3 yr when it was positive and significant. The mean genomic relationship between training and test sets was negative for all 6 yr (Supplemental Table S6). These results suggest that greater similarity of selection material to available historical data would be needed for this strategy to be generally effective.

### 3.5 Genetic gain predictions

Using the prediction abilities of each GEM program and the heritabilities from the IDV models, we calculated relative genetic gain from GS and phenotypic selection (Table 3). For example, the expected response to a single generation of GS on unphenotyped lines vs. direct phenotypic evaluation and selection within the Iowa SS group for grain yield is as follows:

$$\frac{R_{\mathrm{GS}}}{R_{\mathrm{phenotypic}}}=\frac{\mathrm{PA}}{{\hat{h}}^{2}}=\frac{0.75}{0.59}=1.27$$

The relative efficiency of genomic to phenotypic selection ranged from 0.95 to 2.58 across groups, programs, and traits (Table 3), demonstrating that GS has the potential to improve genetic gain generally in the GEM program. This opens many avenues for applying GS and improving the rate of genetic gain within the GEM project. Genomic selection can replace 1 yr of field testing, reducing the breeding cycle time by 1.0–1.5 yr depending on whether winter nurseries are used. Alternatively, GS can be used as a prescreen so that the quality of the material being placed in first‐year testing is enhanced. Lastly, since genotyping costs can be less than the cost of replicated yield trial testing, resources can be shifted from the field to the lab to increase the size of the front end of the breeding pipeline thereby screening more populations and lines in GS and increasing the selection intensity over the whole program.

These relative genetic gains assume that GS is implemented within the same set of biparental crosses that were directly evaluated in the topcross trials, that is, at the same point in the breeding cycle. In reality, training data from prior breeding populations in prior years and seasons are used to predict future breeding populations. This will result in lower prediction ability, similar to the prediction abilities estimated for hold‐one‐year‐out cross‐validation, but in this case, GS is still a very powerful tool, even at lower prediction ability, because it can be implemented in the time that it takes to genotype a sample, in off‐seasons, between seasons, and in a prescreening generation before direct phenotypic selection is even possible (Lorenz et al., 2011; Bernardo, 2020).

### 3.6 Effect of GS on diversity

The primary objective of the GEM project is to diversify the genetic base of the maize crop in the United States; therefore, GS will be useful in the context of this project if its use does not eliminate a substantial proportion of the exotic germplasm in the breeding populations. The high degree of linkage disequilibrium within these populations may hinder the recovery of favorable exotic alleles in linkage blocks dominated by unfavorable alleles. Therefore, we estimated the effect of GS on the proportion of exotic germplasm retained in breeding lines by computing the mean genomic relationships between each GEM line and their parental lines grouped into expired PVP temperate, GEM release line, and tropical parental groups. We included only lines derived from crosses between at least two different groups (19 families).

First, we checked if the mean genomic relationships of lines entered into yield trials agreed with expectations based on their pedigree relationships to the three parental groups (Supplemental File S5). We separated the comparisons into groups based on program and combination of parental groups. We observed higher‐than‐expected genomic relationships between the breeding lines and their tropical founders (ranging from 0.10 to 0.28) for all programs and parental cross combinations except for the crosses between expired PVP and tropical lines in the North Carolina program (0.06 lower than expected genomic relationship; Supplemental File S5). This result suggests that the initial visual selections in GEM nurseries do not eliminate high proportions of exotic germplasm in general.

Next, we checked the correlations between the genomic relationship between each GEM line and its observed grain yield and moisture BLUEs within each family. The correlation between the GEM lines’ yield and similarity to their tropical ancestor was significant in only two families from the Iowa program where it ranged from *r* = −0.33 to −0.34 (Supplemental Table S7). These negative correlations suggest selection for higher yield potential could potentially limit the introgression of exotic germplasm in some families. We also found significant positive correlations (*r* = 0.23 to 0.37) between grain moisture BLUEs and similarity to tropical parental groups within two different Iowa families (Supplemental Table S7). When the correlation between tropical parent relationships and GBLUPs (rather than observed BLUEs) were considered, these unfavorable trends were stronger for both traits (Supplemental Table S8). Significant negative correlations were observed with yield GBLUPs in four families (*r* = −0.31 to −0.49) and significant positive correlations with moisture GBLUPs were observed in five families (*r* = 0.28 to 0.44).

**FIGURE 6:**
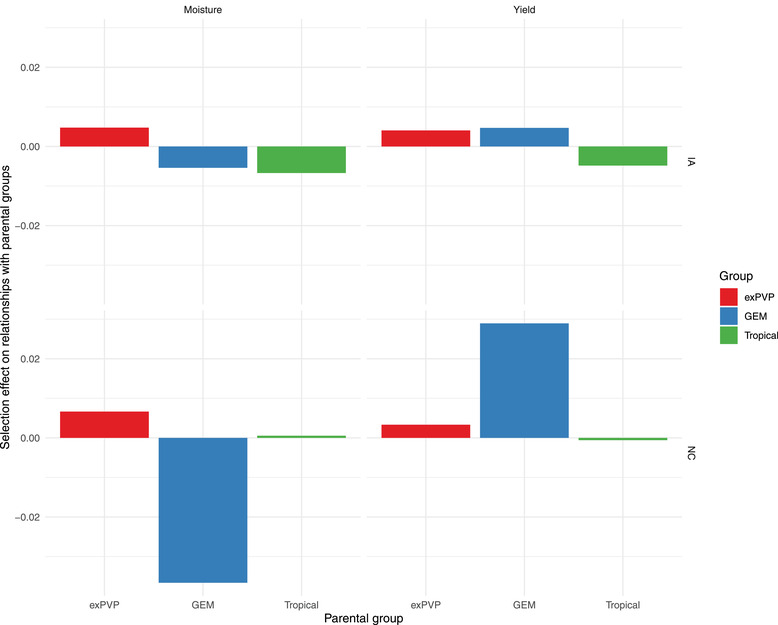
Effect of selection on genomic relationships between Germplasm Enhancement of Maize (GEM) breeding lines and parental germplasm groups. The mean genomic relationship coefficients between each GEM breeding line and each of three parental germplasm groups (expired plant variety protection (PVP) temperate, GEM released line, and Tropical) were computed. The mean relationships between the best 10% of lines based on genomic best linear unbiased predictors (higher yield or lower moisture) and parental groups within each program and heterotic group were also calculated. The selection effect on genomic relationships was estimated as the difference between the mean parental relationships of the selected lines compared with all lines

The unfavorable correlations between the similarity of GEM breeding lines to their tropical parents and their yield and moisture genomic predictions suggest that GS be practiced with care to avoid eliminating too much exotic germplasm. However, the availability of genomic data on the breeding lines permits monitoring of the diversity retained in selected lines. To simulate the effect of selection of the “best” 10% of lines based on their GBLUPs for either yield or moisture within programs and heterotic groups on diversity, we measured the change in genomic relationships between selected lines and the parental groups. Encouragingly, the genomic relationships between the selected lines and tropical parents decreased by no more than 0.01 compared with the unselected set (Figure 6). This result suggests that, overall, GS can be implemented with care in the GEM project to select higher yielding lines that can contribute novel exotic genetic diversity to commercial breeding programs. We also note that the tropical parents used in these breeding crosses were inbreds; we would expect a stronger unfavorable relationship between progeny trait breeding values and relationship to tropical parents sampled from open‐pollinated landrace collections.

### 3.7 Recommendations and future efforts

Combining genomic information with GEM topcross yield data resulted in GS models with strong prediction ability within programs, suggesting that GS can be used to replace first‐year yield trials, thus speeding the delivery of finished GEM releases. The GEM program's limited yield trial resources can be reserved for lines which have first been screened using GS. Genomic information could also be used to monitor the introgression of exotic germplasm to ensure that is not being eliminated at unacceptable rates because of selection. Finally, genomic data can be used to optimize the numbers of lines from each GEM family that are placed in yield trials to test a greater diversity of genetic backgrounds. Our results suggest that distinct prediction models specific to each combination of program and heterotic group should be used. Prediction ability decreases as genetic differentiation between training and selection groups increases (Windhausen et al., 2012; Lorenz & Smith, 2015; Rogers & Holland, 2022). Exchanging breeding germplasm and testing common hybrids between the programs may help improve connectivity in the future. Training set optimization should also be considered as another avenue toward improving prediction abilities (Rincent et al., 2012; Isidro et al., 2015; Akdemir & Isidro‐Sánchez, 2019).

Future efforts should be devoted to updating training sets to closely match current selection candidates. Continued support from Bayer to implement GS for GEM by genotyping and imputing a set of GEM parents and progeny each year as an in‐kind donation will further aid in generating this training set for future model updates. Currently, the GEM project relies on proprietary testers that are owned by GEM cooperators, which permit evaluation of GEM lines in combination with elite commercial germplasm. However, proprietary testers cannot be genotyped, preventing precise characterization of specific combining ability effects in GS models. Shifting to expired PVP or other publicly available lines for use as testers in the future would permit inclusion of specific combining ability effects in genomic prediction models and perhaps identify optimal hybrid combinations for both testing and commercialization earlier in the breeding process.

The GEM programs in Iowa and North Carolina aim at two different target levels for incorporation of exotic germplasm: 25 and 50%, respectively. It would be useful to future breeding efforts to perform a more expansive study of the GEM lines released over the project's lifetime; such a study could provide a better picture of how well these targets are being met, elucidate how GEM is broadening the U.S. maize germplasm pool and where important gaps might be in that base, and provide information about how selection for adaptation to temperate environments affects different regions of the genome.

## 4 CONCLUSIONS

This study was a pilot investigation of incorporation of GS into the GEM project. The results are encouraging, and the GEM programs in Iowa and North Carolina can expect to observe improvements in genetic gain from implementation of GS within their programs while maintaining substantial amounts of exotic germplasm in advanced lines. As of now, because the two programs have little overlap in breeding material, the GS model for one program will not be useful in making selections in the other. Because of this, we recommend that moving forward, cooperators in GS for GEM generate breeding values for each program from their respective model for use by GEM breeders in making selections.

## AUTHOR CONTRIBUTIONS

Anna R. Rogers: Data curation; Formal analysis; Investigation; Software; Writing – original draft; Writing – review & editing. Yang Bian: Formal analysis; Investigation; Methodology; Software; Writing – review & editing. Matthew Krakowsky: Data curation; Investigation; Writing – review & editing. David Peters: Data curation; Investigation; Writing – review & editing. Clint Turnbull: Investigation; Project administration; Resources; Writing – review & editing. Paul Nelson: Conceptualization; Project administration; Resources; Supervision; Writing – review & editing. James B. Holland: Formal analysis; Investigation; Software; Writing – original draft; Writing – review & editing.

## CONFLICT OF INTEREST

The authors declare no conflict of interest.

## Supporting information

Supplemental Figure S1 is a histogram of the environment yield BLUPs for the IA GEM program.

Supplemental Figure S2 is a histogram of the environment yield BLUPs for the NC GEM program.

Supplemental Figure S3 displays boxplots of moisture mean values by origin within the IA data set.

Supplemental Figure S4 displays boxplots of moisture mean values by origin within the NC data set.

Supplemental Table S1 contains the list of released GEM lines, their pedigree, country and race of exotic parent, and year of release.

Supplemental Table S2 contains best linear unbiased estimators (BLUEs) for grain yield and grain moisture of each GEM line, averaged over environments and testers, for IA GEM program.

Supplemental Table S3 contains best linear unbiased estimators (BLUEs) for grain yield and grain moisture of each GEM line, averaged over environments and testers, for NC GEM program.

Supplemental Table S4 contains yield prediction ability summaries by training and test set groupings.

Supplemental Table S5 contains moisture prediction ability summaries by training and test set groupings.

Supplemental Table S6 contains prediction ability values for grain yield and moisture when all data from one year of the IA program is held out as a test set.

Supplemental Table S7 contains within‐family prediction ability correlation estimates between the yield or moisture BLUE of a line and its similarity to each of the three parental germplasm groups.

Supplemental Table S8 contains within‐family prediction ability correlation estimates between the yield or moisture GBLUP of a line and its similarity to each of the three parental germplasm groups.

## Data Availability

Supplemental data files are available online (https://doi.org/10.5061/dryad.g1jwstqss). Supplemental figures, tables, R markdown scripts, and knitted R markdown files are available online (https://doi.org/10.5281/zenodo.7150254).
